# Proteomic and Phenotypic Studies of Mycoplasma pneumoniae Revealed Macrolide-Resistant Mutation (A2063G) Associated Changes in Protein Composition and Pathogenicity of Type I Strains

**DOI:** 10.1128/spectrum.04613-22

**Published:** 2023-06-28

**Authors:** Zhikun Zhang, Haiwei Dou, Qing Yuan, Dawei Shi, Ruijie Wan, Peng Tu, Deli Xin, Shuilong Guo

**Affiliations:** a Department of Science and Technology, Beijing Friendship Hospital, Capital Medical University, Beijing, China; b Beijing Tropical Medicine Research Institute, Beijing Friendship Hospital, Capital Medical University, Beijing, China; c Department of Pathogenic Biology, School of Basic Medicine Southwest Medical University, Luzhou, China; University Paris-Saclay, AP-HP Hospital Antoine Béclère, Service de Microbiologie, Institute for Integrative Biology of the Cell (I2BC), CEA, CNRS

**Keywords:** *Mycoplasma pneumoniae*, proteomics, A2063G, proliferation, adhesion

## Abstract

Mycoplasma pneumoniae (MP) is an important respiratory pathogen, the prevalence of macrolide-resistant MP (mainly containing A2063G mutation in 23S rRNA) increased in recent years. Epidemiological studies suggest a higher prevalence of type I resistant (IR) strains than corresponding sensitive (IS/IIS) strains, but not type II resistant (IIR) strains. Here, we aimed to analyze the factors underlying the altered prevalence of IR strains. First, proteomic analyses exhibit the protein compositions were type specific, while more differential proteins were detected between IS and IR (227) than IIS and IIR strains (81). mRNA level detection suggested posttranscriptional regulation of these differential proteins. Differential protein-related phenotypic changes were also detected: (i) P1 abundance was different between genotypes (I < II, IR < IS), the adhesion of MPs showed accordance to P1 abundance within IS and IIS strains; (ii) type I, especially IR, strains had a higher proliferation rate, which is potentially associated with differential proteins participating in glycolysis and one carbon pool metabolisms; (iii) A549 cells infected with IR strains had lower activity of caspase-3 and higher levels IL-8, but the differences were not significant between groups (*P* > 0.05). Correlations of P1 abundance to caspase-3 activity and proliferation rate to the level of IL-8 were obtained. These results suggest changes in protein composition influenced the pathogenicity of MP, especially in IR strains, which may impact the prevalence of MP strains of different genotypes.

**IMPORTANCE** The prevalence of macrolide-resistant MPs increased the difficulty in treatment of MP infections and posed potential threats to children's health. Epidemiological studies showed a high prevalence of IR-resistant strains (mainly A2063G in 23S rRNA) in these years. However, the trigger mechanisms for this phenomenon are not clear. In this paper, proteomic and phenotypic studies suggest that IR strains have reduced levels of multiple adhesion proteins and increased proliferation rate, which may lead to higher transmission rate of IR strains in the population. This suggests that we should pay attention to the prevalence of IR strains.

## INTRODUCTION

Mycoplasma pneumoniae (MP) is one of the leading pathogens for community-acquired pneumonia (CAP); it can take up to 40% of the causative agent in pediatric CAP (1). MP is naturally resistant to β-lactam antibiotics because of the absence of a cell wall. Macrolides are the first choice of treatment for MP infection in children. However, the prevalence of macrolide-resistant MP increased the difficulties in treatment of Mycoplasma pneumoniae pneumonia (MPP) in children (2, 3). Epidemiological studies suggest that the isolation rate of macrolide-resistant MP strains increased these years. In Southeast Asia, the resistance rate can reach up to 80% in some regions (4, 5). Along with the increase in macrolide resistance correlations between resistant strains and genotype were reported in several reports. That is the isolation rate of type I clinically resistant strains was significantly higher than that of sensitive strains, which was not observed in type II strains (6 to 9). This indicates the pathogenicity of type I strains may be influenced by drug-resistant mutations.

The two subtypes of MP have been described with significant sequence differences in P1, which is the major adhesion protein of MP (10). Comparative genomic analyses suggest about 1,500 nucleotide differences between the two MP subtypes (11). P1 is located at the tip terminal of MP as a part of the adhesion organelle; it is also the antigenic epitope of MP (12). In addition to P1, the adhesion organelle of MP contains a variety of adhesion molecules, including P30, P65, P200, HMW1, HMW2, and HMW3. The adhesion molecules have been shown to play important roles in the infection and pathogenicity of MP (10). Epidemiological studies suggest a genotype shift in the prevalence of two subtypes of MP (8). This suggests antigenic differences between the two MP subtypes and the importance of adhesion organelle in infection (8).

The adhesion organelle is involved in the pathogenicity of MP. First, adhesion is critical to MP infection. Due to the limitation of genome size MP is defective in carbon source and nucleic acid metabolism (13). During adherent infection, MP can accelerate the proliferation rate by feeding on the host cells nutrition. Then, adherent infection can evade the clearance of respiratory cilia and cause direct damage to host cells (14). Meanwhile, MP can produce superoxide and the community-acquired respiratory distress syndrome (CARDS) toxin, thus promoting cell damage during infection (15). Finally, MP adhesion is also engaged in the inflammatory induction process. MP lipoproteins can be recognized by TLR2/6 and TLR2/1 heterodimers, leading to the regulation of proinflammatory cytokines such as TNF-α, IL-1β, IL-4, and IL-6 (16, 17). MP infection has been shown to promote the expression of IL-8 and IL1β in lung epithelial cells (18), and the induction of IL-4 in mast cells (19, 20). Both cytokines are involved in the inflammatory response in the lung during MPP and show relevance to the development of severe MPP (21).

The main prevalent macrolide-resistant MP contains the A2063G mutation, which is located at the central loop in domain V of 23S rRNA. In E. coli the 2063 site is located at the exit nascent peptide of the 23S rRNA ribosome (22), and this domain was proved as the binding site for macrolide antibiotics (23). *In vitro*, A2063G mutation results in resistance to macrolides, especially azithromycin, suggesting the conformation of this domain is influenced (24). Further, the loop of domain V was shown with peptidyl transferase activity, which is involved in the formation of peptide bonds during peptide chain extension (25). To maintain the normal function of ribosomes, 23S rRNA is relatively conservative among species. The drug resistance mutation in MP is mainly A→G, which leads to the conformational change of amino residue (-NH2). Whether A2063G mutation leads to functional changes of the ribosome and shows correlation to the protein synthesis and pathogenicity of MP still needs to be characterized.

In this paper, resistant (A2063G) and sensitive MP strains of two genotypes were selected based on epidemiology study. Protein composition and pathogenic phenotypes of these strains were detected. The results suggested more resistance-related changes in protein composition of type I strains. Protein composition-related changes in pathogenic phenotypes were also detected in type I resistant strains. These results help clarify the prevalence of type I resistant strains.

## RESULTS

### Differential proteins between groups are type specific.

**FIG 1:**
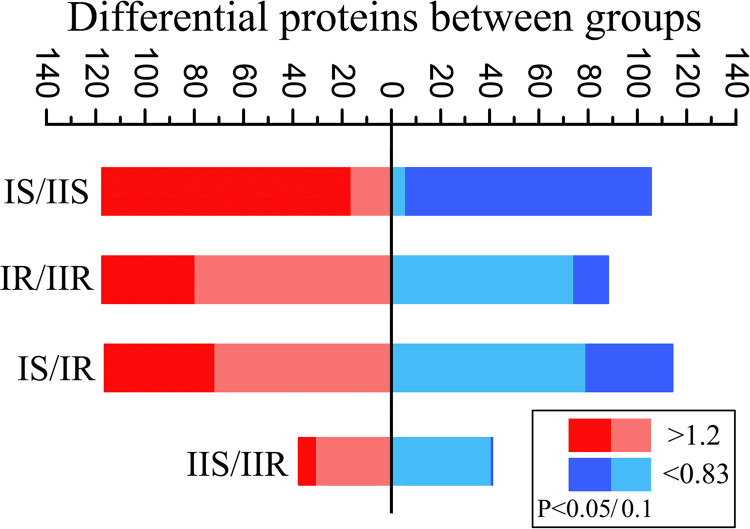
Statistical differential proteins between groups. The differential proteins were obtained by comparison between groups (IS, type I sensitive strains; IIS, type II sensitive strains; IR, type I resistant strains; and IIR: type II resistant strains). A ratio greater than 1.2 was considered high and a ratio less than 0.83 was considered low. A *P* value <0.05 and 0.1 was considered significant.

**FIG 2:**
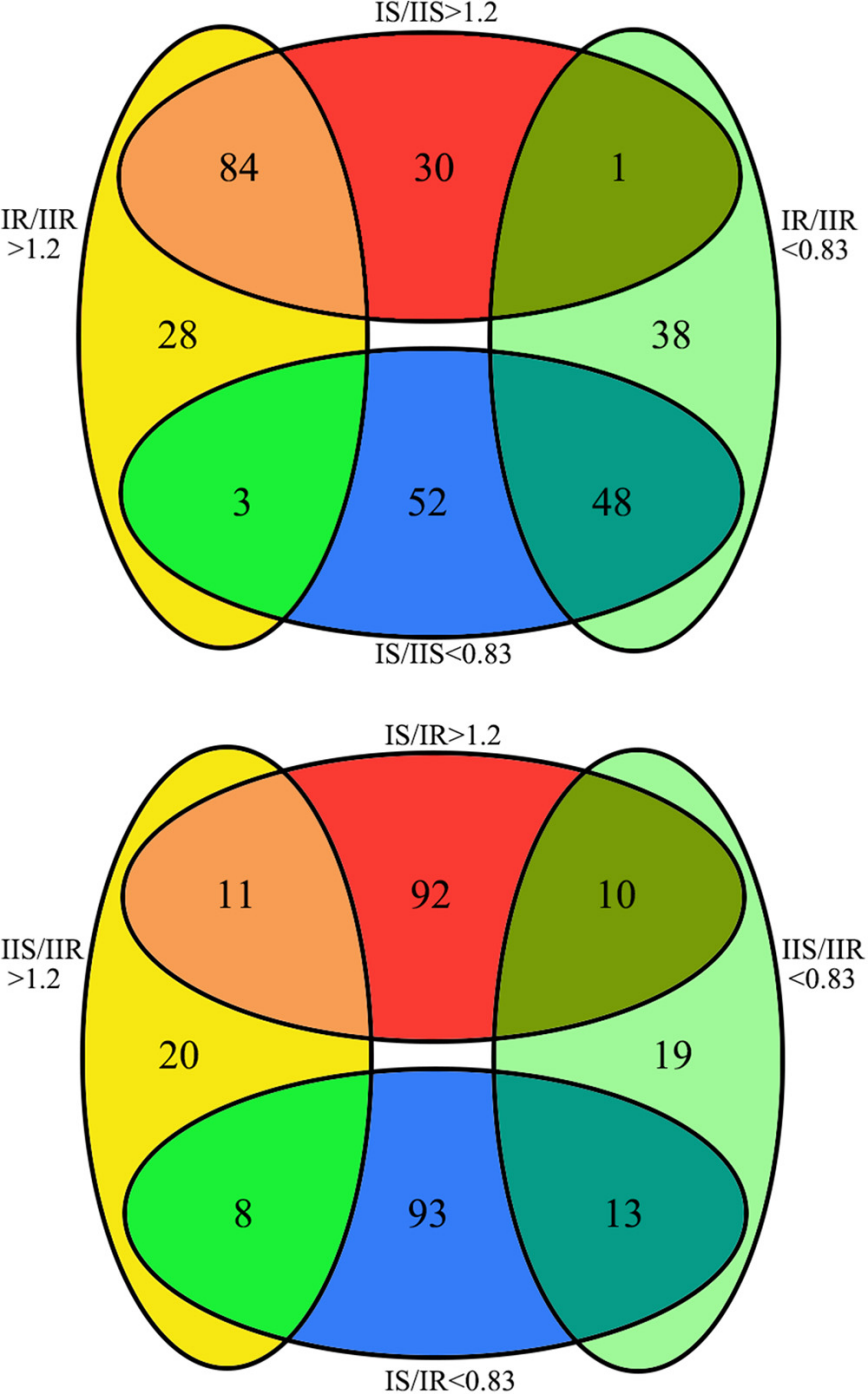
Venn diagram of differential proteins. The overlapping regions represent the number of identical proteins between groups (IS, IIS, IR, and IIR strains).

Through TMT-labeled detection, a total of 651 proteins were detected among all strains. Protein abundance analyses reveal the protein composition of MP is genotype specific. Between type I and type II strains, a total of 115 upregulated and 103 downregulated proteins were identified in sensitive strains (IS versus IIS), a total of 115 upregulated and 87 downregulated proteins were identified in resistant strains (IR versus IIR) (Fig. 1). The differential proteins between resistant and sensitive strains share 84 identical upregulated proteins and 48 identical downregulated proteins (Fig. 2). Within genotypes, 113 upregulated and 114 downregulated proteins were identified in type I strains (IS versus IR), and 39 upregulated and 42 downregulated proteins were identified in type II strains (IIS versus IIR) (Fig. 1). Few identical proteins were observed between the differential proteins of two genotypes (Fig. 2). Volcano plot analysis revealed higher fold changes in the differential proteins between two genotypes than resistance-related ones (see Fig. S1 in the supplemental material). These results suggest different protein composition in MP of two genotypes and more resistance-related changes in protein composition in type I strains.

### Function analysis of differential proteins.

By functional analysis, all the differential proteins were mainly classified to ribosome and protein synthesis, DNA replication, carbohydrate metabolism, membrane transporters, and virulence. As shown in Table 1, more proteins related to ribosome and protein synthesis and carbohydrate metabolism were obtained between groups followed by the DNA replication and membrane transporters (detailed protein information is listed in Table S3). P1 adhesion was lower in type I strains than in type II strains. More adhesion-related proteins (P1, P30, HMW1, HMW2, and HMW3) were higher in IS strains than in IR strains. P200 adhesion and CARDS were higher in type I strains than in type II strains. The level of CARDS was higher in IS strains than in IR strains. The difference in protein composition may influence the adhesion, proliferation, and pathogenicity of MP strains, which needs further validation.

**TABLE 1:** Function statistics of differential proteins between different subtypes of MP

Gene function	IR/iIR >1.2	IR/iIR <0.83	Is/iIS >1.2	Is/iIS <0.833	IIS/iIR >1.2	IIS/iIR <0.83	Is/IR >1.2	Is/IR <0.83
Ribosome and protein synthesis	18	8	16	16	1	3	6	16
DNA	5	3	4	3	0	1	4	3
Carbohydrate metabolism	9	10	9	11	1	2	17	15
Membrane transport	6	2	5	2	1	0	5	2
Adhesions and CARDS	P200; CARDS	P1; P30	P200; CARDS	P1	0	0	P1; HMW1, 2, 3; P30 CARDS	0
Others	26	31	33	29	9	16	24	32

### Most differential proteins are caused by posttranscriptional regulation.

**FIG 3:**
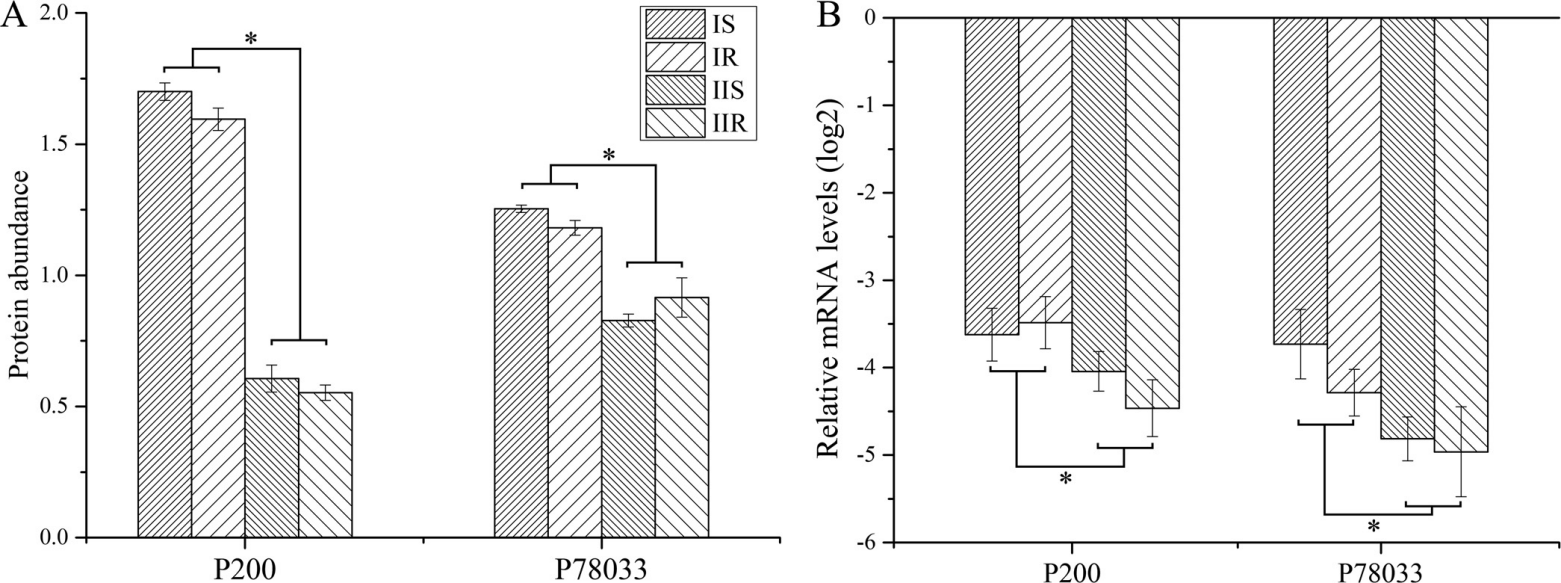
Abundance and mRNA levels of differential proteins. (A) Protein abundance was obtained from TMT results. (B) The mRNA levels were detected by quantitative PCR, the *y* axis indicates the relative expression compared to 23S rRNA (log_2_).

To analyze the causes of different protein compositions in MP strains, 27 differential proteins (Table S2) were selected for mRNA quantitative detection. The results showed that the mRNA levels of P200 (adhesion) and P78033 (glucose-6-phosphate isomerase) were significantly different between type I and type II strains (*P* < 0.05). Meanwhile, the protein abundance of these two proteins correlated with their mRNA levels (Fig. 3). On the other hand, no significant differences were detected in the mRNA levels of the remaining 25 proteins (not shown), suggesting differences in these proteins are not regulated at transcript levels. P200 and P78033 are only differential proteins between type I and type II strains (Fig. 3, Table S3). This indicated the differential proteins between IS and IR strains are mainly triggered by posttranscriptional regulation.

### Cell adhesion shows correlation to P1 abundance within sensitive groups.

**FIG 4:**
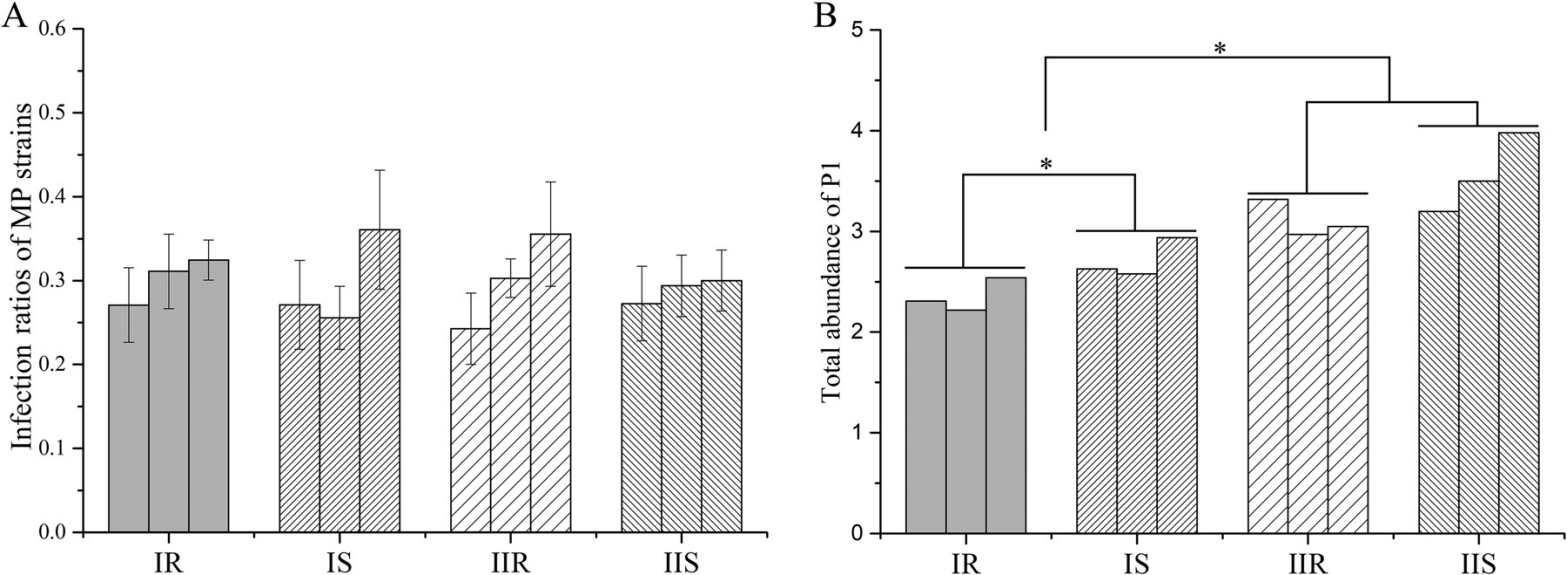
MP adhesion rate and P1 abundance. (A) Adhesion rate of MP strains to A549 cells: MP was incubated with A549 cells at a ratio of 1:5 for 6 h. The infection rate was derived by comparing the amount of MP on the cell surface to the total MP in the volume. (B) P1 protein abundances derived from TMT results.

To verify the changes in differential proteins related functions, phenotype studies of the 12 strains were tested. For the differences in adhesion proteins, the adhesion activities of these strains to A549 cells were detected. As indicated, the average adhesion rate of each group within 6 h was between 28.87% and 30.12%, and there was no significant difference between groups (Fig. 4A). This indicates the adhesion rate to human cells is not significantly influenced. However, a potential correlation (*P* < 0.01) between the adhesion rate and P1 abundance was observed within IS and IIS groups (Fig. 4B, Table 2).

**TABLE 2:** Correlation analysis of pathogenic phenotypes^*a*^

Items	Caspase-3	IL1β	IL8	P1	-CCK8	Proliferation	Adhesion	CARDS
Caspase-3		0.664**	−0.105	0.531*	−0.197	−0.291	0	−0.343
IL1β	C		0.406	0.133	−0.254	0.004	0.364	−0.343
IL8	C	ACD		−0.559*	0.127	0.630*	−0.189	0.126
P1	AD		b		−0.028	−0.592*	−0.091	−0.706**
-CCK8	Ac	Bc	c	A		−0.071	−0.141	−0.120
Proliferation	A	Bd	D	A	Ab		−0.424	0.291
Adhesion	D	A	Ab	BD		c		0.098
CARDS	a	d	bd	aBc	a	ad	B	

a Correlations between all strains were indicated with Spearman rho values. Correlations within groups (A/a: IR, B/b: IS, C/c: IIR, D/d: IIS) were indicated with letters (positive/negative correlation). An asterisk (*) indicates correlation is significant under 0.05 (unilateral). Two asterisks (**) and letters indicate correlation is significant under 0.01 (unilateral).

### Multiplication of MP correlates with the changes in carbohydrate metabolic proteins.

**FIG 5:**
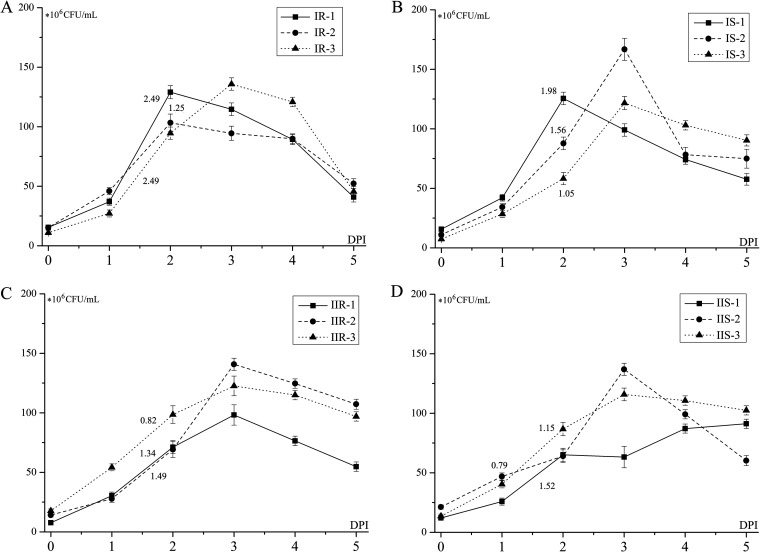
Growth curves of MP strains of different genotypes: (A) type I resistant strains (IR); (B) type I sensitive strains (IS); (C) type II resistant strains (IIR); (D) type II sensitive strains (IIS). The *x* axis represents the days postincubation (DPI), 0 days represents the original inoculum volume, and the values marked on the lines represent the multiplication coefficient ([48–24 h]/24 h MP load) of the strains between 1 and 2 days of each strain.

**FIG 6:**
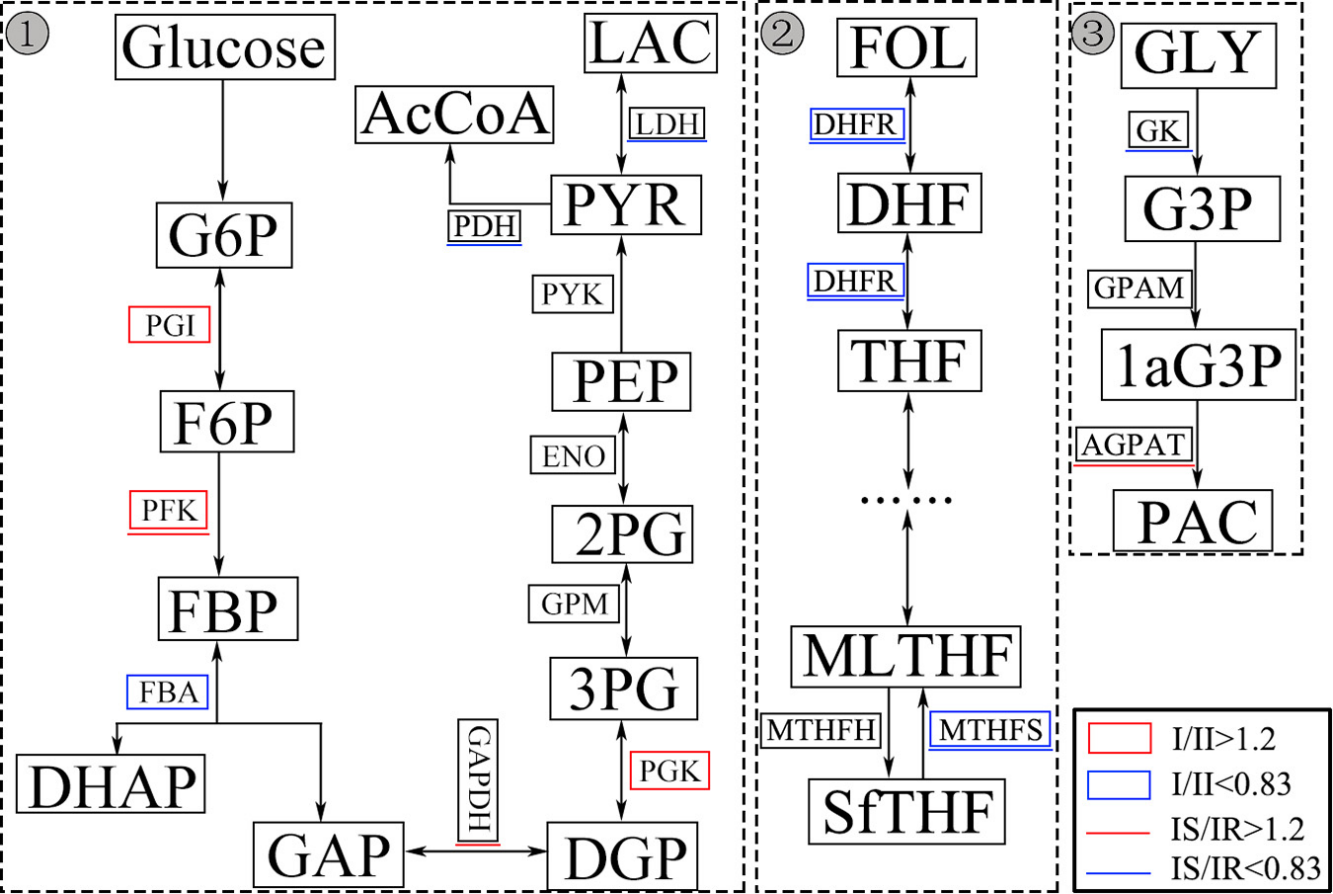
Functional enzymes involved in glycolysis (1), one carbon pool of folate (2), and glycerol (3) metabolism pathways. Differential proteins between type I and type II strains are marked with colored boxes, differences between type I resistant and sensitive strains are marked with colored underlines. Red represents high levels and blue represents low levels in type I or IS strains.

The growth curves of MP strains were tested in SP4 medium, the total MP in culture mediums were tested every 24 h according to its proliferation rate. As shown in Fig. 5, all the strains peak at 48 to 72 h postincubation. Type I strains tend to have a higher 24-h multiplication coefficient “(48h to 24 h)/24 h MP load” at the logarithmic growth phase (24 to 48 h), among which IR1 and IR3 got the highest multiplication coefficients (Fig. 5). Carbohydrate metabolic pathway analysis showed that type I strains had higher levels of 1-phosphofructokinase (PFK) and glucose-6-phosphate isomerase (PGI) than type II strains. Meanwhile, IR strains had a higher level of dihydrofolate reductase (DHFR), tetrahydrofolate synthase (MTHFS), and glycerol kinase (GK) compared to IS strains (Fig. 6). These differential proteins involved in carbohydrate metabolic may affect the glycolysis, folate, and glycerol metabolism, thus influencing the proliferation of MP.

### P1 abundance and proliferation show correlations to pathogenicity of MP.

**FIG 7:**
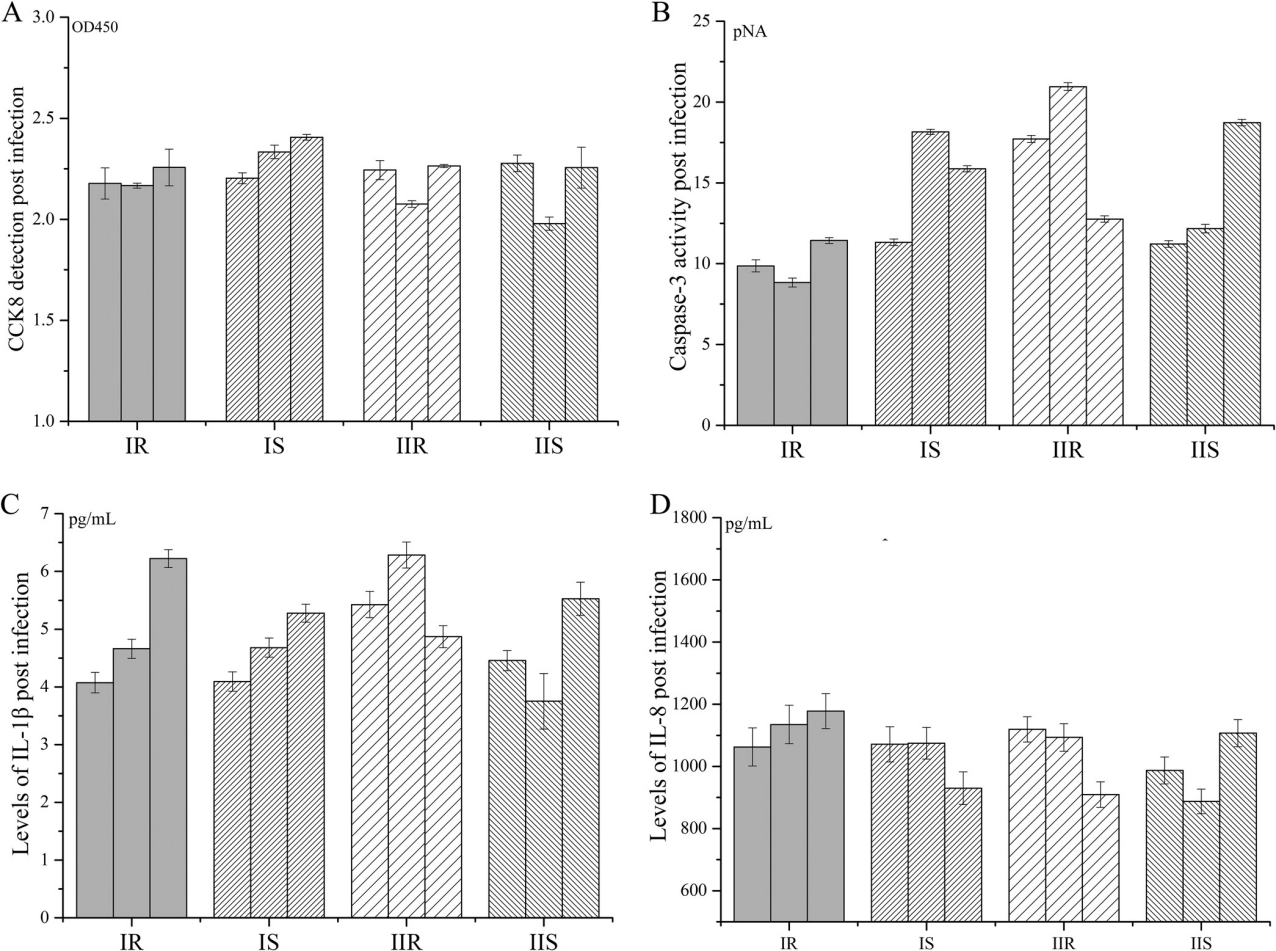
*In vitro* pathogenicity detection. MP strains were incubated with A549 cells (MP: A549 = 1: 5). (A) Cell viability (CCK8), (B) apoptosis (caspase-3 activity), (C) intracellular IL-1β levels, and (D) extracellular IL-8 levels at 24 h postincubation.

Finally, the pathogenicity of these strains was detected *in vitro*. The results showed that the levels of cellular activity (CCK8) and expression of IL-1β were similar among all groups of strains at 24h postinfection. Lower levels of apoptosis (caspase-3 activity) and higher levels of IL-8 were detected in cells infected with IR strains, but the difference between groups was not statistically significant (*P* > 0.05) (Fig. 7). Correlation analysis revealed that caspase-3 activity post MP infection correlated with the level of IL-1β and P1 abundance; the level of IL-8 correlated with proliferation rate but negatively correlated with P1 abundance; P1 abundance show negative correlations to CARDS abundance and proliferation rate (Table 2). Within groups the correlations between pathogenic-related factors are more complex, only consistent correlations between the level of IL-1β and IL-8 were observed. These suggest the pathogenicity of MP is influenced by multiple factors. The differences in P1 abundance and proliferation may impact the apoptosis and cytokine induction of infected cells, and then influence the pathogenicity of IR strains.

## DISCUSSION

As indicated in the present study, A2063G mutation-related differential proteins were mainly detected in type I strains rather than type II strains. This may relate to the difference in ribosomal protein composition of different genotypes. According to the TMT results, the abundance of 30S ribosomal protein S7 and 50S ribosomal protein L4 was higher in type I strains than in type II strains, while 30S ribosomal protein S2 was lower than in type II strains (Table S3). Among the ribosomal proteins, L4 has been shown to locate near the peptidyl transferase region, suggesting different conformations in the peptidyl transferase region between type I and type II strains (26), and the ribosomal structures of type I and type II strains may act differently to A2063G mutation. Our results suggest A2063G mutation has a greater impact in type I strains with elevated 30S ribosomal proteins S12 and S2 in IR strains, which may influence the protein synthesis functions of IR strains (27, 28). Differences in ribosomal protein composition in IR strains were also reported in a recent study of single strain proteomics analysis (29). Changes in protein synthesis and transmembrane transport-related proteins that associated with drug resistance were also reported (29). Through analysis of the differential proteins in the present study, an elevated proportion of acidic and basic amino acids were detected in the higher proteins rather than lower proteins in IR strains compared to IS strains (not shown). This suggests a potential effect of A2063G mutation to the peptidyl transferase activity of IR strains. Sequence analyses also reveal high ratios of acidic and basic amino acids in P1 proteins (12, 30). Amino acid composition of P1 is helpful in analyzing the correlation between A2063G mutation and changes in adhesion proteins in IR strains. However, the specific role of A2063G mutation in ribosomal functions still needs to be elucidated.

Further, our results suggest correlations between proliferation and P1 abundance to the pathogenicity of MP. Clinically, a correlation between MP load in the lower respiratory tract and the severity of pulmonary disease in MPP patients has been reported (31, 32). The induction of IL-8 in lung cells by MP infection *in vitro* is also dose-dependent (33). These are consistent with the correlation between proliferation rate and the level of IL-8 in the present study. A quick proliferation of MP in the lung may lead to quick activation of the innate immune response in the lung (34). On the other hand, correlations between cytadherence of MP and inflammatory responses have been reported. MP strains treated with P1 antibodies or with low P1 expression decreased the induction of proinflammatory cytokines (17, 35). Lack of HMW1 and P30 or P40 and P90 were also reported with reduced induction of IL-1β (36). Meanwhile, the correlation between P1 abundance and caspase-3 activity may relate to the difference in cell damage during adhesion. As shown in Table 1, IR strains had lower P1, HMW1, HMW2, HMW3, and P30 protein compositions in apical adhesion structures than IS strains. Although IR and IS strains have similar infection rates in infection experiments, they may have different adhesion strengths (37). The reduction of all these adhesion-related proteins may result in decreased toxicity and sliding ability of IR strains (38, 39). In addition, though the proliferation rate was measured *in vitro*, it can provide insights into the reported correlation between P1 and DNA content (40). The negative correlation between proliferation and P1 abundance may relate to natural selection during transmissions (41). In general, we hypothesize the patients infected with IR strains may have a higher load of MP in the lung, which facilitates the transmission of IR strains.

Carbohydrate metabolism was considered a main factor influencing the proliferation rate of IR strains. PFK1 is a major rate-limiting enzyme of glycolysis. Higher level of PFK1 may increase the level of glycolysis in type I strains (42). Folate metabolism may also serve as a factor limiting the proliferation of IS strains compared to IR strains. Lower dihydrofolate reductase content in IS strains may affect the formation of tetrahydrofolate, which in turn affects the synthesis of nucleic acids and amino acids as well as cell growth and division (43). In addition, protein synthesis may also influence the proliferation rate of type I strains, for multiple tRNA ligases were detected as higher in type I strains (Table S3). Due to the small genome size, metabolic pathways of MP are susceptible to environmental factors. Glucose, nucleic acid, and protein levels in *in vitro* culture systems have been shown to correlate with MP proliferation (13). Therefore, differences in carbohydrate metabolism and protein synthesis may directly affect the proliferation rate of MP. However, MP has been shown to regulate carbohydrate metabolism and protein expression patterns through the HPr kinase/phosphorylase during infection (44). The impact of these differential proteins on the proliferation and virulence of MP during infection *in vivo* still needs to be further elucidated. On the other hand, this study exhibited a higher level of CARDS in IS strains, which was reported as higher in type II strains and common between IS and IR strains (29, 45). This difference may relate to the strains that were used for proteomics analysis. Epidemiological studies have revealed genotypic changes and geographical differences of MP in these years (46), and the expression of CARDS may be different from genotypes. In addition, the strains used in this study are both clinical strains less than two *in vitro* passages. The expression of CARDS may decrease in the strains that adapted to *in vitro* culture systems, as CARDS does not promote the proliferation of MP *in vitro*.

In conclusion, this study exhibited a distinctive protein composition of IR strains. The low abundance of adhesion proteins and high proliferation rate of IR strains correlated with the changes in pathogenicity. This indicates a potential relevance of A2063G mutation to the prevalence of IR strains.

## MATERIALS AND METHODS

### MP strains and cells.

Twelve clinical strains less than two *in vitro* passages that were isolated in Beijing, China during 2009 to 2018 were employed in this study, including three strains each of the type I resistant (IR), type I sensitive (IS), type II resistant (IIR), and type II sensitive (IIS) strains (Table S1). MP strains were cultured in SP4 medium in 37°C incubator with 5% CO_2_. After incubation, all strains were plated on an SP4 agar plate after serial dilution, the CFU was obtained under a microscope. All cultures were diluted to about 10^7^ CFU/mL and stored at −80°C. Basal epithelial human lung adenocarcinoma A549 cells were cultured with RPMI 1640 supplemented with 10% fetal bovine serum (FBS) in a 37°C incubator with 5% CO_2_.

### Proteomics detection.

The stored MP was gradually expand cultured in SP4 medium to a final volume of 200 mL. MP cells were suspended and collected by centrifugation at 8,000 *g* for 30 min at 4°C, followed by two washes with 15 mL PBS. The collected cells were transported on dry ice for proteomics detection (APT, China). Protein detection of MP strains was performed using Tandem Mass Tags (TMT)-labeled mass spectrometry. In brief, total proteins were extracted with SDT (4% SDS, 1 mM DTT, 100 mM Tris-HCl, pH 7.6) buffer and quantified with the BCA protein assay kit (Bio-Rad, USA). Proteins were digested by trypsin according to filter-aided sample preparation (FASP) procedure (47). The digest peptides of each sample were desalted on C18 cartridges (Empore, Sigma), concentrated by vacuum centrifugation, and reconstituted in 40 μL of 0.1% (vol/vol) formic acid. Then, 100 μg peptide mixture of each sample was labeled using TMT reagent according to the manufacturer’s instructions (Thermo Scientific). The labeled peptide mixtures were then applied in strong cation exchange fractionation and LC-MS/MS analysis.

The MS raw data for each sample were searched using the MASCOT engine (Matrix Science, London, UK; version 2.2) embedded into Proteome Discoverer 1.4 software for identification and quantitation analysis. The obtained data after a database search was compared between groups for analysis of differential proteins. An abundance fold change (FC) >1.2 or <0.83 between groups with a *P* value < 0.1 (*t* test) was considered up- or downregulated. Functional annotation of all differential proteins was performed using Blast2Go (https://www.blast2go.com/) software; the proteins were annotated by the KEGG pathway database for protein resolution (48).

### mRNA detection.

According to the proteomics results, 27 proteins with significant differences between groups were selected for the quantitative detection of their coding mRNAs using 23S rRNA as reference, the primers are listed in Table S2. In brief, the 12 strains were cultured in SP4 medium for 48 to 72 h, then the MP cells were collected by centrifugation at 8,000 *g* for 30 min at 4°C. Total RNA of the cells were extracted with TRIzol regent (Invitrogen), the extracted RNA was reverse transcribed (Universal RT-PCR kit, Solarbio) and detected by fluorescent quantitative PCR method (UltraSYBR RT-qPCR kit, CWbio).

### Adhesion and growth curves.

The adhesion of MP strains was measured on A549 cells. Six-well cell plates were inoculated with 5*10^5^ A549 cells per well and incubated for 24 h. After incubation, the medium was replaced with fresh medium containing MP (MP: A549 = 1:5), using 3 wells for each strain. Cells were incubated for 6 h and the medium was discarded. Then, the cells were washed twice with 2 mL PBS, cells were collected by trypsin digestion, grounded, and suspended using PBS. The lysates were inoculated on SP4 agar plates after gradient dilution and incubated for 3 to 4 days for colony counting. The original medium was also diluted and inoculated on SP4 agar plates for colony counting. The adhesion rate of each strain was calculated by comparing the amount of MP on the cells to the total amount of MP.

The stored MP strain was 1:10 mixed with fresh SP4 medium, the volumes were subsequently transferred to 5 mL tubes, each tube 1 mL with 5 tubes per strain. The tubes were incubated in a 37°C incubator with 5% CO_2_. The MP in 1 tube of each strain was collected every 24 h, the original solution and all cultures were serially diluted and spread on SP4 agar plates for colony counting. All the colony counting plates were conducted with three replicates for each dilution.

### Cytotoxicity assay.

A549 cells were inoculated in a 96-well plate with 1*10^4^ cells per well and incubated for 24 h. The medium was replaced with fresh medium containing MP (MP: A549 = 1:5) and further incubated for 24 h with six replicates of each strain. Then the volumes were changed with RPMI 1640 containing CCK8 detection reagent (BBI, China) and incubated at 37°C for 2 h. The absorbance values (OD450) of each well were measured by a microplate reader.

Six-well plates were inoculated with 5*10^5^ A549 cells per well and incubated for 24 h. The medium was replaced with fresh medium containing MP (MP: A549 = 1:5) and incubated for another 24 h. After incubation, the medium and cells were collected separately. The cells were resuspended with RPMI 1640 and used for detection of IL-1β (Solarbio, China), the medium was used for detection of IL-8 (USCN, China). A replicate experiment was conducted and the cells were collected for detection of caspase-3 activity (Solarbio, China). All the samples were detected by three replicate wells in each reagent kit.

### Statistical analysis.

All statistical analyses in this paper were performed using IBM SPSS Statistics for Windows, version 20.0 (IBM, Armonk, NY, USA). Data of each strain or group was indicated as mean ± standard deviation (SD). The Student’s *t* test was used for comparison between groups. Probability (P) values <0.05 were considered statistically significant. Spearman correlation coefficient rho was used as the index of correlation; *P* < 0.05 (unilateral) was considered statistically significant.

### Data availability.

All data supporting the research findings of this study are included within the article and in the supplemental material. Raw data of Tandem Mass Tags (TMT)-labeled mass spectrometry have been deposited in iProX integrated proteome resources (https://proteomecentral.proteomexchange.org/cgi/GetDataset?ID=PXD041114).
