# Supplementary material for: Proteomic and Phenotypic Studies of Mycoplasma pneumoniae Revealed Macrolide-Resistant Mutation (A2063G) Associated Changes in Protein Composition and Pathogenicity of Type I Strains
Source: Microbiol Spectr. 2023 Jun 28;11(4):e04613-22. doi: 10.1128/spectrum.04613-22 (PMC10434051; doi:10.1128/spectrum.04613-22)
Supplement: Supplemental file 1 — Supplemental material. Download spectrum.04613-22-s0001.pdf, PDF file, 0.2 MB [file spectrum.04613-22-s0001.pdf]

Table S1 Mycoplasma pneumoniae strains used in this study. All the strains were isolated from hospitals in Beijing China, and store in Beijing Friendship Hospital.

Storage number	BS303	CYM219	BS296	1037C	317B	794B	BS362	CYM267	ETBX364	MT54	MT214	MT208
Experiment number	IIR	IIR	IIR	IR	IR	IR	IS	IS	IS	IIS	IIS	IIS
Isolation (Years)	2016	2016	2016	2016	2009	2014	2017	2017	2018	2016	2016	2016

Table S2: Primers for fluorescent quantitative PCR detection.

Proteins	Forward primer	Reverse primer
P75545	AAGTACCTCAAGACCGCAAGATT	GCTTCAGCCATCTTATGCGTATC
P75579	GCACACTGTTTGGAGTGAAAAGT	TGCTGGTGCTTTGTTCTAAGTTG
P75590	TTCGTACCATGCAAGACGAAAAC	TTTTTGCTGTTCGCGGAACTTAT
P78024	TTTAAAGCTAAGCAAACGACGGG	GCAGTTAGTGGTTTTTGGACCTC
A0A0H3DLH4	ACAGTAATGGTAATGCGGTGAGT	GTCAAACGTTACCTCTACCCCTT
P75122	TGATACTGAAACCAAAACCGTGC	GCTTTTTGCCCTTACCAATCACT
P78033	GGTTCTTATACTGGCATTCGTGC	AGTGTGCGACCAGATTTAGAAGT
P75095	GCGTTGCTTTTAGTCTTCCAACT	ACCATTGTTAGCGAGGTCAAGAT
P75264	ACGATGGTAGTCCCGTATTTAGC	TACCTACTTCAAACTGGTGGCTC
A0A0H3DLK2	CGCTGATTGGGTGGTACTTTAAC	ACGTTTAAGTGATTTTACCCCGC
A0A0H3DLU7	TTCATTACCGCTTTTGTCGCTTT	TAGCTACTTGACGGGCTAATTCC
A0A0H3DJS0	GGTGGGGAAGAAGATGGTATTGT	GCACTAAGGGTAAGTTAGTCGCT
A0A0H3DLA5	CTTTACCGCAGGGTTGAATTACC	AACCAGTGTTGACCTTACACCTT
A0A7U4SLC2	CGGTTTTGTAATGTGAACGGGAA	CCAAGTGGCAATTAAGTCAGCAA
P75039	TCAGTATGACTTTGTGGCGGTAA	GGTTCTGAATCCCATCAGTTCCT
A0A7U4NSG3	GTGGGTGTTTGAACGCAAGATTA	CCAAAATGAACCGTATCCTGAGC
P75554	ACTGACAAACCCTCTTTTCAGGT	ATCATCCCGAAGATAATGCCCAA
P75188	TTAAGGGTGTCCAAAAGACCAGT	ACGGTTTCTAATGGTGGGATCAA
A0A0H3DLF3	TCACAAGGAAATTAACCTAGACAACCT	TAAACGACGCGCACGATTAGTA
A0A0H3DLK2	GAAGTAGCACAAAACAACGGTCA	ACTTCTCCAAGCGTTCAATTTCG
A0A0H3DLE9	TTTTGAGCGGACTAAGCTAGAGG	GGTACGTGGATCAAAGGTGTTTT
P200	AAGTGGTACGATGGGGAATTTGA	CCTTAGCATCGGAATCGGAAAAC
CARD	CAAACTGGTTACAGCTGGGATTG	CACCTTTAACTGCTGGTTGTCAG
P30	CAGCACAACAATACCGAACTGAC	CTAAAACCAAAGCAACCAGCAAC
HWM1	AACTCCTACCGTTACAACCAGTC	TTTCGGTTCGCTTACTCGTCATA
HWM3	AGATCCAAAAGAGCTATCCCGAC	TGCCATCTTGGTCGTAAAAGGTA
HWM2	TCAGCTATCGGAAAACGTGAAGA	ATGTCTTTTCCCCCACTAGCATT
P1	ACTTCCACAATAACCCCGATTGA	ACCGTATTTAACCTTGTCGGGAA

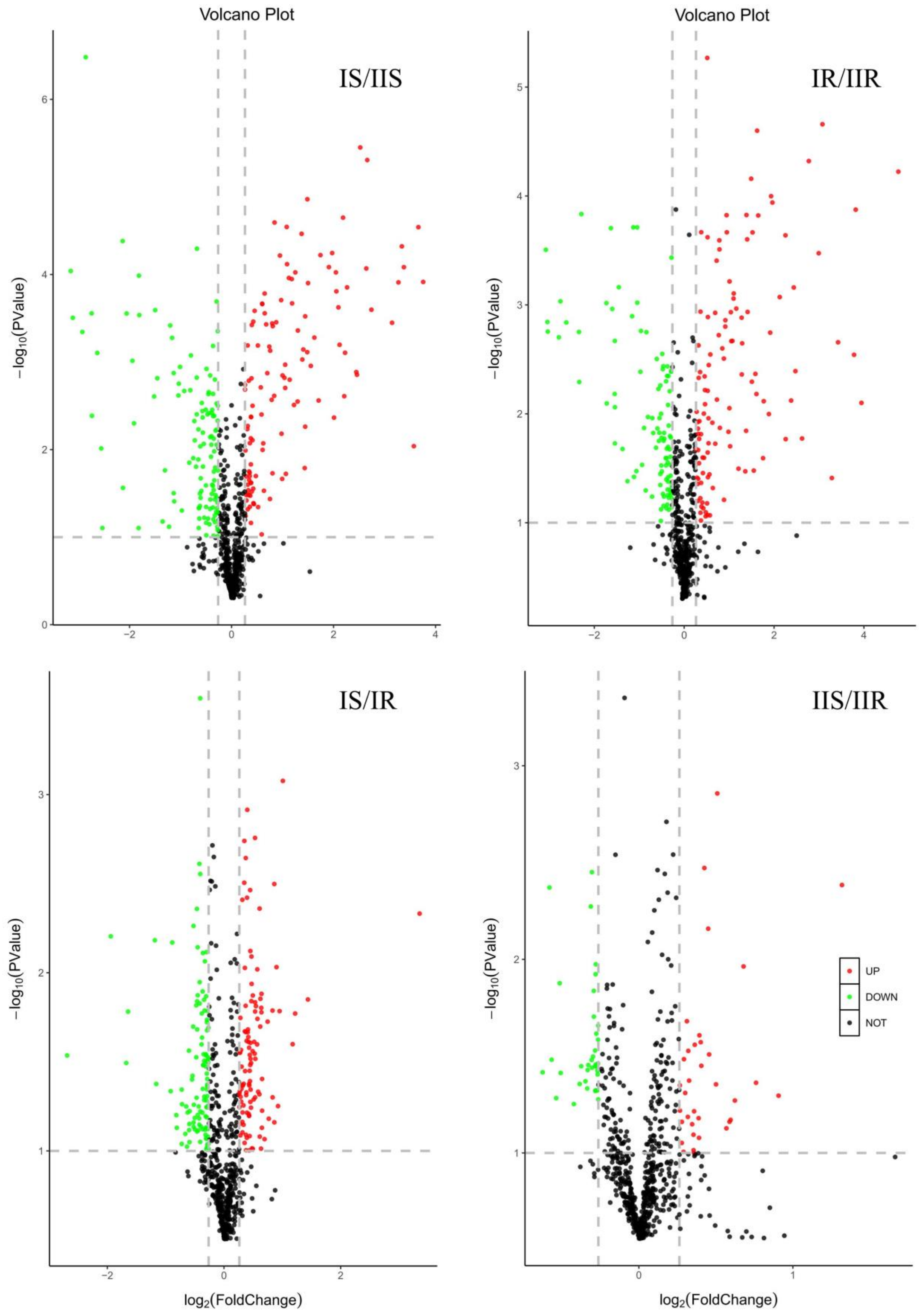

Figure S1: Volcano plot of differential proteins: the differential proteins were analyzed between by fold change >1.2 or <0.83 between groups (IS: type I sensitive strains, IIS: type II sensitive strains, IR: type I resistant strains, IIR: type II resistant strains) with a P value<0.1.
